# Femoral tunnel-interference screw divergence in anterior cruciate ligament reconstruction using bone-patellar tendon-bone graft: A comparison of two techniques

**DOI:** 10.4103/0019-5413.80045

**Published:** 2011

**Authors:** Vivek Pandey, Kiran Acharya, Sharath Rao, Sripathi Rao

**Affiliations:** Department of Orthopaedics, Kasturba Medical College, Manipal, Karnataka, India

**Keywords:** Anterior cruciate ligament, screw divergence, femoral tunnel, interference screw

## Abstract

**Background:**

Interference screw is a popular fixation device used to rigidly fix bone-patellar tendon-bone (B-PT-B) graft both in femoral and tibial tunnels in anterior cruciate ligament (ACL) reconstruction. Parallel placement of screw is difficult in transtibially drilled femoral tunnel but always desired as it affects pullout strength of the graft. Commonly, interference screw into the femoral tunnel is inserted through the anteromedial (AM) or accessory AM portal. These portals are not-in-line with the transtibially drilled femoral tunnel. Furthermore, these portals increase the divergence of the interference screw in the femoral tunnel. We hypothesized that interference screw placement through patellar tendon (PT) portal (through donor defect) in transtibially drilled femoral tunnel can be less divergent. We report the prospective randomized study to investigate the difference of divergence of interference screw placed through PT portal and AM portal and its clinical relevance.

**Materials and Methods:**

Forty-one patients underwent femoral tunnel B-PT-B graft fixation through AM portal (group 1) and other 41 (group 2) through PT portal. Femoral tunnel-interference screw divergence was measured on postoperative digital lateral X-rays. Ha’s method was used to grade divergence. The clinical outcome was assessed by postoperative intervention knee documentation committee grading (IKDC) and Lysholm score at 2 years followup.

**Results:**

Mean tunnel-screw divergence in sagittal plane through AM portal was 13.38° (95% CI: 12.34-14.41) and through PT portal was 7.20° (95% CI: 6.25-8.16) (*P*<0.0001). In AM portal group, 82.9% patients had divergence in either grade 3 or 4 category, whereas in PT portal group, 82.9% patients were in grade 1 or 2 category (*P*<0.0001). Mean Lysholm score were 92.8 and 94.5 at two-year follow-up in both groups which were statistically not significant. The International knee documentation committee grades of patients in both groups were similar and had no statistical significance.

**Conclusion:**

Femoral interference screw placement through the PT portal leads to significantly less screw divergence as compared with screw placement through the AM portal. However, this difference in divergence is not reflected in clinical outcome.

## INTRODUCTION

Bone-patellar tendon-bone (B-PT-B) graft is a reliable method for anterior cruciate ligament (ACL) reconstruction.^1^–^3^ After graft fixation in the tunnel, bone block healing occurs in various animal models by 8 to 12 weeks.^4^ Rigid fixation is mandatory for reliable healing of bone block with tunnel, so that accelerated rehabilitation protocol can be allowed in the early phases.^4^–^6^ Various options are available for fixation of the graft in the tibial and femoral tunnels.^7^–^11^ Interference screw is a reliable and frequently used method for graft fixation.^12^–^18^ It also provides excellent fixation and pullout strength to the graft. Various factors affect the pullout strength of the graft: bone block size, quality of bone, gap between bone block and tunnel, screw diameter and length, and the angle between screw and bone block (parallel or divergent).^19^–^25^ Parallel placement of screw with respect to femoral and tibial tunnel is desired as it is one of the factors which affect pullout strength of the graft.^21^ Biomechanical and clinical ramifications of a divergent interference screw in the bone tunnel (tibial or femoral) is now well established and is one of the key factors in the success of the B-PT-B graft.^19^–^21^ It is easy to place the interference screw parallel to the bone block in the tibial tunnel as direction of drilling and placement of the interference screw are same. Commonly, the interference screw in a transtibially drilled femoral tunnel is usually placed through the anteromedial (AM) portal or accessory AM portal over a guide wire. However, this is not-in-line with the transtibially drilled femoral tunnel and can lead to screw divergence. If the divergence is more than 15° between interference screw and bone block, the pullout strength of the graft decreases sharply affecting the graft-tunnel healing, pullout strength, and the clinical outcome.^20^,^21^,^26^–^29^ We hypothesized that patellar tendon (PT) portal through donor defect is more in the line with femoral tunnel and hence would minimize the tunnel-interference screw divergence.

The primary aim of this prospective randomized comparative study was to investigate whether an interference screw placed through the PT portal shows less divergence as compared with a screw placed through the AM portal. The secondary aim was to investigate the difference in the clinical outcome between the two modes of screw placement.

## MATERIALS AND METHODS

Eighty two consecutive patients who underwent ACL reconstruction by between January 2006 and January 2010 by B-PT-B graft were included in this study. The block randomization method was used with allocation ratio of 1:1. After proper informed consent, ACL reconstruction by B-PT-B graft for unstable ACL-deficient knee was performed with standard technique. Of 82, 41 patients underwent graft fixation in femoral tunnel by AM portal (group 1), while other 41 patients through PT portal (group 2) through donor defect. All ACL reconstructions were performed by a single surgeon. Postoperatively, tunnel-screw divergence was measured (degree) in sagittal plane on a digital lateral X-ray of the knee with the help of software embedded in GE-PACS (GE Pathspeed Web 2.0, USA). Angle was measured in degrees between the longitudinal axis of tunnel and interference screw by an experienced radiologist on two separate occasions. The radiologist was blinded for the technique used (AM or PT portal). Screw divergence was classified into four grades, as described by Kwon Ick Ha *et al*.^30^ Grade 1 (no divergence) were patients with 0° to 5° divergence; grade 2 were 5° to 10° (Mild); grade 3 were 10° to 15° (moderate); and grade 4 were greater than 15° (severe). The clinical outcome was assessed by postoperative International Knee Documentation Committee (IKDC) grading and Lysholm score at twoyear follow-up.

### Statistical analysis

Data were entered in statistical package of social sciences 13.0 (SPSS, Chicago, USA) and analyzed. The difference in angle measured was analyzed for any statistical significance. Intraobserver variation was analyzed at two different occasions. Mean angle of divergence of both groups was calculated. Means of two groups were compared using independent T test. Association between grades of divergence between two groups was done using chi square test. *P* value <0.05 was considered as significant.

### Operative procedure

**Figure 1:**
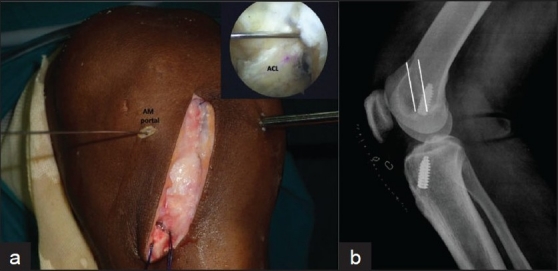
(a) Guide wire through AM portal showing divergence in the arthroscopic image (inset) in the left knee. (b) Lateral X-ray of knee showing interference screw divergent to femoral tunnel (parallel white lines depict outline of femoral tunnel)

**Figure 2:**
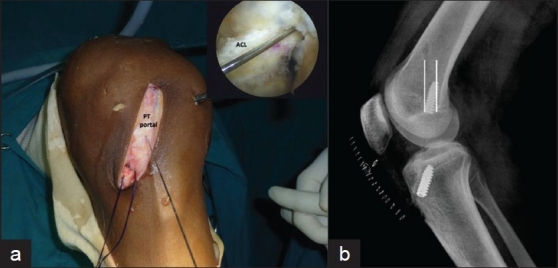
(a) Guide wire through PT portal showing parallel wire in the arthroscopic image (inset) in the left knee. (b) Lateral X-ray of knee showing interference screw near parallel to femoral tunnel (parallel white lines depict outline of femoral tunnel)

After spinal or general anesthesia, diagnostic arthroscopy was performed under tourniquet through standard AM and anterolateral (AL) portal. All patients underwent single incision ACL reconstruction using B-PT-B graft. A 9 mm wide × 25 mm long bone plug was harvested from patella and tibial tuberosity by standard technique. After performing diagnostic arthroscopy, the intercondylar notch and lateral wall were prepared. The tibial tunnel was prepared by serial drilling from 6 to 9 mm cannulated reamers over a guide wire exiting 6 mm anterior to the anterior fibers of posterior cruciate ligament, just medial to medial tibial spine in the posterior footprint of torn ACL fibers. With the knee at 80 to 90° of flexion, femoral tunnel was drilled over a guide wire which was kept 7 mm anterior to posterior edge of lateral femoral condyle at 10:30 o’clock in right knee and 1:30 o’clock in left knee using a femoral offset of 6 mm. Bicortical drilling was done with 4-mm cannulated reamer. Then, 30 mm long femoral tunnel was drilled serially with 7- to 9-mm cannulated reamers. Then, femoral tunnel was notched supero-laterally through the portal, which was decided for passing the interference screw. B-PT-B graft was passed into the femoral tunnel through the tibial tunnel over a beath pin which exited from anterolateral aspect of thigh. The cancellous portion of graft was facing anterolaterally. Knee was flexed to 110° and a guide wire advanced into the notched portion of tunnel to minimize divergence through the selected portal, AM or PT portal, through the donor defect [Figures 1 and 2]. To achieve graft fixation in femoral tunnel, titanium interference screw of appropriate length and diameter was inserted over the guide wire using the selected portal (according to randomization) into the femoral tunnel. Again, guide wire was kept parallel to graft in tibial tunnel and graft was fixed with interference screw keeping the knee at 30° flexion with force directed posteriorly onto the shin of tibia. After the ACL reconstruction, Lachman and Pivot shift test were performed to assess the adequacy of fixation. Postoperatively, anteroposterior (AP) and lateral digital X-ray of knee were taken using GE-PACS system. Patient was started on standard accelerated ACL rehabilitation protocol from the next day.

## RESULT

There were 71 males and 11 females. Forty-one patients underwent femoral tunnel fixation by titanium interference screw through AM portal (group 1) and other 41 patients through PT portal (group 2). The intraobserver variation of tunnel-screw angle was statistically not significant. The mean tunnel-screw divergence in sagittal plane through AM portal was 13.38° (95% CI: 12.34-14.41) and through PT portal through donor defect was 7.20° (95% CI: 6.25-8.16), which is illustrated in Table 1.The difference between the means was found to be statistically significant (*P*<0.0001) using independent sample test. Grade of divergence in both groups are illustrated in Table 2. It was observed that in AM portal group, 82.9% of the patients had divergence in either grade 3 or 4 category, whereas in PT portal group, 82.9% of the patients were in grade 1 or 2 category [Table 2]. Chi square test applied between the two groups was statistically significant (*P*<0.0001). Two-year follow-up of patients in both groups had average postoperative Lysholm score of 92.8 and 94.5, respectively. The differences between Lysholm score were statistically not significant, indicating that there was no difference in clinical outcome of the two groups. The IKDC grades of patients in both groups are mentioned in Table 3. They were either grade A or B (normal or near normal). Irrespective of divergence grades, no patient had poor outcome. There is no difference between the clinical outcomes of both groups at the two-year follow-up.

**Table 1:** Mean angle of interference screw-femoral tunnel divergence between two groups

Group/Portal	Mean angle of tunnel-screw divergence (degrees)	95% confidence interval
Group 1 Anteromedial portal N = 41	13.38	12.34-14.41
Group 2 Patellar tendon portal N = 41	7.20	6.25-8.16

Independent sample test for equality of means (*P*<0.0001)

**Table 2:** Grades of interference screw-femoral tunnel divergence between two groups

	Grade of divergence			
	Grade 1	Grade 2	Grade 3	Grade 4
Group/Portal	(0-5°)	(5-10°)	(10-15°)	(>15°)
Group 1 Anteromedial portal (% within portal)	0 (0%)	7 (17.1%)	24 (58.5%)	10 (24.4%)
Group 2 Patellar tendon portal (% within portal)	11 (26.8%)	23 (56.1%)	06 (14.6%)	01 (2.4%)

Chi square test: (*P*<0.0001)

**Table 3:** IKDC grading of knees at two-year follow-up in two different groups with respect to the grade of interference screw-tunnel divergence

	Grade of divergence 1		Grade of divergence 2		Grade of divergence 3		Grade of divergence 4	
	IKDC A	IKDC B	IKDC A	IKDC B	IKDC A	IKDC B	IKDC A	IKDC B
Group 1 Anteromedial portal (N = 41)	0	0	6	1	22	2	8	2
Group 2 Patellar tendon portal (N = 41)	10	1	21	2	5	1	1	1

IKDC: Intervention knee documentation committee

## DISCUSSION

Interference screw fixation of B-PT-B graft in femoral and tibial tunnels still remains a popular method of fixation.^1^–^3^ Recently established trends of accelerated rehabilitation protocol for ACL reconstruction demand that the fixation of graft in tunnel should be strong and rigid.^4^–^6^ Feagin was the first one to note that screw divergence can lead to loss of fixation strength.^18^ Screw divergence inside the femoral tunnel can lead to inappropriate fixation of bony graft and decreased pullout strength. Jomha *et al*. reported that pullout strength decreases with increasing divergence (0°, 10°, 20°, and 30°) in his study on 20 porcine knees.^21^ He reported that maximal fixation is observed at 10° of divergence and decreases as angle increases. He also reported that there is not much difference in neighboring groups. However, the difference was significant between far groups (0° and 30°). In another model using 90 porcine knees and 5 human cadaveric knees, Fulkerson *et al*. reported that divergence more than 30° leads to loss of fixation and significantly decreased pullout strength.^20^ Lemos *et al*. in a bovine model reported that divergence less than 15° did not affect fixation characteristics.^27^ T hough many authors have reported varying acceptable degrees of divergence, the surgical objective is always to place the screw in the femoral tunnel as parallel to the graft as possible.^20^,^21^,^27^ Divergence is observed more in sagittal than in coronal plane. Recently, Ninomiya *et al*. assessed the fixation strength of hamstring graft in femoral tunnel and effect of screw divergence in coronal place.^31^ He too concluded that divergence greater than 15° significantly compromises the pullout strength.

Several techniques have been described by various authors to minimize the divergence of interference screw in femoral tunnel. Brodie *et al*. described modified Paulos technique to minimize the divergence.^32^ He used the tibial tunnel to push the femoral screw into the femoral tunnel. However, it needs a larger tibial tunnel drilling which can weaken the tibial fixation. Schroeder, using flexible single use Straight shot graft passer (DePuy Orthopaedic Technology, Tracy, CA), developed a technique using the tibial tunnel to push the femoral screw.^33^ However, it also dilates the tibial tunnel, needs a special instrument and the screw driver can damage the graft in the tibial tunnel. Rodin and Levy described the use of intraoperative fluoroscopy to minimize femoral interference screw divergence during ACL reconstruction.^34^ But this technique although increasing the operative time, adds the risk of radiation exposure.

Recently, a newer technique has been developed by Chan and Wang where they also have used the tibial tunnel for femoral screw insertion while keeping the graft in AM portal which prevents any possibility of graft laceration while screw insertion.^35^ Furthermore, the tibial tunnel need not to be over dilated to facilitate screw entry. Hence, graft fixation is not compromised and it does not need any special instruments.

To our knowledge, there are no published studies in English literature comparing two methods of interference screw placement in femoral tunnel and divergence. Our results prove that acceptable levels of divergence can be achieved by using PT portal through donor defect with correct knee positioning. The mean divergence remained 7.2° which is within normal limits of divergence and does not affect pullout strength of graft. The technique does not require the making of an additional portal (accessory AM portal) or special instrument. The fear of damaging infrapatellar fat pad by traversing interference screw seems to be exaggerated. While inserting the interference screw, no undue pressure should be applied as it can force the fat pad into the joint and can obscure the vision temporarily. Gentle screwing action is sufficient to negotiate the interference screw through fat pad. Divergence within 30° is said to be acceptable as far as clinical result is concerned. However, the more in line PT portal through donor defect is much effective in reducing the divergence and keeps it to the minimum level. Also, to our knowledge, there are no published studies which compared the divergence of interference screw by different methods with respect to the clinical result in patients. In our study, the average postoperative Lysholm score were similar in both groups 97.14 and 98.02 (AM and PT portal, respectively), indicating that though there were radiological differences between both groups; there was no difference in clinical outcome. The IKDC objective grades were also similar in both groups, indicating that though there is angle difference between the two groups, it may not lead to any clinical difference as long as the divergence angle is within acceptable limits.

Interference screw divergence in the femoral tunnel can also lead to bending of intrafemoral tunnel portion of guide wire. If the guide wire bends, it becomes difficult to pull out the guide wire. In order to accomplish that, sometimes it might be necessary to remove the interference screw and reinsert, which weakens the interference fit. So, using an appropriate portal (PT) can minimize the guide wire bending as it avoids excess divergence. Rarely, divergence can also lead to cortical penetration causing blow out or graft fracture in femoral tunnel.

The limitation of this study is that only sagittal plane divergence has been evaluated. It is difficult to accurately outline the femoral tunnel in the coronal plane in AP view of X-ray. However, a randomized study with larger sample size is desired to study the relation between multiplanar divergence (using computed tomography scan) and clinical outcome using these two portals.

Femoral interference screw placement through the PT defect provides significantly less screw divergence as compared with screw placement through the AM portal. However, this difference in divergence is not reflected in the clinical outcome.
